# Practical Support from Fathers and Grandmothers Is Associated with Lower Levels of Breastfeeding in the UK Millennium Cohort Study

**DOI:** 10.1371/journal.pone.0133547

**Published:** 2015-07-20

**Authors:** Emily H. Emmott, Ruth Mace

**Affiliations:** Department of Anthropology, University College London, London, England; NIH, UNITED STATES

## Abstract

Mothers face trade-offs between infant care and subsistence/economic activities. In traditional populations, allomothers such as fathers and grandmothers support mothers with young infants, allowing them to reduce labour activities and focus on breastfeeding. Similarly, the positive impact of social support on breastfeeding has been highlighted in developed populations. However, these studies have generally focused on emotional support from fathers, peers and healthcare professionals. Given the availability of formula milk in developed populations, an evolutionary anthropological perspective highlights that practical support, unlike emotional support, may have negative associations with breastfeeding by enabling substitution of maternal care. Other kin, mainly grandmothers, may also be important allomothers influencing maternal breastfeeding levels. Here we explore the associations between different types of social support mothers receive from fathers/grandmothers and breastfeeding in the UK Millennium Cohort Study. We find frequent grandmother contact and father’s parenting involvement are both associated with lower levels of breastfeeding, suggesting a negative relationship between practical support and breastfeeding. In contrast, father presence, potentially capturing emotional support, is associated with greater breastfeeding initiation. Our findings suggest that practical support and emotional support functions differently, and practical support may not encourage breastfeeding in developed populations.

## Introduction

In traditional, high-fertility high-mortality populations, breastfeeding is an obligate maternal investment behaviour which is essential for child survival. Mothers incur energetic costs[1,2] and reduced fecundity[3] to provide the only adequate food source available for infants. Breastfeeding is often incompatible with subsistence and other labour activities, meaning breastfeeding mothers can struggle to provide enough resources for themselves and their children[4,5]. Consequently, breastfeeding mothers in traditional contexts are dependent on allomothers such as fathers and grandmothers for support, especially in terms of resource provisioning[4–6]. Such support could increase fertility and reduce infant mortality, thus enhancing reproductive success[7,8]. Several evolutionary anthropologists have argued that allomother support is an obligate human trait which accompanies breastfeeding, with paternal support facilitated by the evolution of pair-bonding[4,5] and grandmother support facilitated by the evolution of menopause[6]. The significance of such allomothers in traditional contexts, as well as in our evolutionary history, has been highlighted in the proposal that humans evolved to be cooperative breeders[9], where successful reproduction and childrearing is contingent on the support from multiple helpers.

In developed, low-fertility low-mortality populations, the relatively novel environments people face bring into question whether allomothering functions in a similar manner as traditional contexts. Specifically regarding breastfeeding, mothers in developed populations experience different trade-offs, with novel costs and benefits surrounding infant feeding. There are some costs of breastfeeding in developed contexts which seem similar to that of traditional populations: Breastfeeding is still energetically costly for the mother, with exclusive breastfeeding estimated to require 400 to 750 kcal/day depending on milk production[1,2]. Further, breastfeeding can clash with maternal activities such as wage labour[10–12]. However, breastfeeding in developed populations is no longer a necessity for child survival. Breastfeeding may be substituted by formula-feeding, which may be associated with greater financial expense, but it introduces the potential for mothers and allomothers to *share* infant feeding and the associated costs. In contrast to traditional populations where practical allomother support for mothers with young infants are more or less constrained to resource provisioning, allomothers in developed populations may be able to help through greater levels of infant caregiving.

With the promotion of formula milk[13], combined with the costs of breastfeeding to mothers, it is not surprising that the availability of evaporated milk and commercial formula in the West was followed by a reduction in breastfeeding rates starting in the 1930s/40s, reaching the lowest levels in the 1970s[13]. However, there is now strong evidence that breastfeeding, compared to formula feeding, is associated with extensive health benefits for children including immunological protection and prevention of infectious disease, reduced risk of asthma and atopy, better cognitive and motor development, as well as reduced risk of obesity[14–22]. Such findings have resulted in the introduction of various pro-breastfeeding policies in the U.S. and Europe, also reflected in WHO’s baby friendly hospital initiative and its recommendation for mothers to breastfeed exclusively for 6 months[21,22].

Western breastfeeding rates have been rising over the last few decades[13,21]. In the UK, breastfeeding initiation rates have risen from 62% in 1990 to 81% in 2010, and any breastfeeding at 6 months has risen from 21% in 1995 to 34% in 2010[23]. However, only 1% of UK mothers achieved the WHO-recommended exclusive breastfeeding for 6 months in 2010[23], and breastfeeding rates in the UK is among the lowest in Europe[21]. In comparison, in 1997, breastfeeding initiation in Sweden was near 100% while exclusive breastfeeding at 6 months was at 42%[21]. Variation in breastfeeding levels are also observed within nations, with factors such as age, ethnicity and socio-economic status associated with different levels of breastfeeding initiation in the UK[23]. With this disparity in breastfeeding levels and the difficulty in meeting the WHO recommendation, there has been great interest surrounding what encourages mothers to breastfeed, with one line of enquiry focusing on social support. If humans still operate as cooperative breeders in developed populations, support from allomothers may facilitate breastfeeding as we observe in traditional populations. Or, given the opportunity to share infant feeding, we could also expect differences in the relationship between allomother support and maternal breastfeeding.

### Social Support and Breastfeeding in Developed Populations

Recognising the maternal costs of breastfeeding, researchers across disciplines have made analogous arguments to evolutionary anthropologists that mothers require social support for successful breastfeeding[24,25]. Such social support has been broadly categorised into two themes[26]: *Emotional/Informational Support* relates to the provisions of supportive information, as well as interactions which improve self-appraisal and self-esteem. *Instrumental/Practical Support* often relates to supportive behaviours such as active assistance and financial support.

The positive impact of support on breastfeeding initiation and duration is claimed to be well established[24,25]. However, a review of the literature points to ambiguity as well as bias surrounding the definition of support. In a systematic review of 34 quasi-randomised controlled trials surrounding social support, support was defined as “Contact with an individual (either professional or volunteer) offering support that is supplementary to standard care (in the form of, for example, appropriate guidance and encouragement) with the purpose of facilitating continued breastfeeding[24].” With this definition, the distinction between the two types of support is not explicit, though the example suggests the focus is on emotional and informational support. It is also common to find that social support is not explicitly defined, though such studies often focus on positive attitudes and encouragements towards breastfeeding[25,27,28], which again overlaps with emotional support. Social pressure to breastfeed may also be treated as a form of social support[25], though it is possible that this reflects emotional coercion rather than support *per se*.

Such ambiguity surrounding the definition of social support means the two different types of support may have different functions and pathways. From an evolutionary anthropological perspective, whether or not you carry out a behaviour is influenced by the social norms relating to that behaviour[29–31], as well as the costs and benefits surrounding that behaviour[30–32]. While feedback is expected between the two pathways, they should be treated as separate entities with independent effects[31]. Whether or not a mother breastfeeds depends on the norms she experiences surrounding breastfeeding, as well as the costs and benefits she incurs from breastfeeding.

Emotional and informational support centres on the transfer and maintenance of pro-breastfeeding attitudes, such as supporting the idea to breastfeed and boosting maternal confidence to do so. This type of social support may be inherently linked to *breastfeeding promotion*, and one could even argue against its conceptualisation as support, given that such information and attitudes can only be supportive if mothers have a desire to breastfeed in the first place. This connection between emotional/informational support and breastfeeding promotion is an important point to highlight, as it is conceivable that breastfeeding promotion primarily affects maternal breastfeeding norms.

In contrast, practical support is likely to have a different pathway, influencing the costs and benefits surrounding breastfeeding. Practical support for mothers such as helping behaviour and financial transfers could encourage breastfeeding if it leads to the substitution of other maternal activities (e.g., substitution of domestic and/or paid work), where mothers are better able to focus on breastfeeding. If so, we would expect both practical and emotional support to deliver the same outcome. Alternatively the availability of practical support may *discourage* breastfeeding: Formula-fed infants are less dependent on mothers for feeding, which may increase opportunities for helpers to provide practical childrearing support. This potential to share the costs of childrearing may serve as an incentive for mothers to formula-feed. Support may, from an evolutionary perspective, also contribute to increased maternal investment in a child by acting as a cue that a much-helped child could help lead to better child outcomes (and therefore lack of allocare may lead to lower maternal investment for the same reason). The lack of studies addressing practical support means that the associations between practical support and maternal breastfeeding behaviours in developed populations are currently unclear.

### Types of Supporters and Breastfeeding in Developed Populations

In evolutionary anthropology, the cross-cultural importance of kin support for childrearing has been highlighted[9,33]. Studies have found that grandmothers are particularly influential allomothers where their presence impacts breastfeeding duration, child survival and maternal fertility in natural fertility and developing populations[4,9,33–35]. However, the majority of studies on social support and breastfeeding in developed populations focus on emotional and informational support from healthcare professionals, fathers, and peers[24,25,28]. Perhaps due to nuclear family norms of the West, investigations into the impact of grandmothers on breastfeeding in developed populations have been scarce.

Of the handful of studies that are available, the results are inconsistent. Some studies find that grandmother support encourages breastfeeding. For instance, in a survey of 123 US mothers with infants and toddlers, mothers reported that greater support from grandmothers and other family members would have encouraged them to breastfeed[36]. In an Australian randomised controlled trial involving 72 mothers attending antenatal breastfeeding classes, mothers in the intervention group who brought a female breastfeeding supporter, often the maternal grandmother, breastfed for longer[37]. In contrast, other studies suggest that social support from grandmothers may in fact discourage breastfeeding. In the US, maternal co-residence with grandparents predicted lower rates of breastfeeding initiation for a disadvantaged sample of households, and shorter duration of breastfeeding for both a disadvantaged and a nationally representative sample of households[38]. Similarly, a study on Brazilian mothers found that daily contact with maternal grandmothers, compared to less frequent contact, had a negative association with breastfeeding duration[39].

Interestingly, the positive associations between grandmother support and breastfeeding seem to centre on emotional and informational support. In contrast, the studies which focus on grandmother contact, potentially capturing practical support and the ability to act as substitute carers at an early age, indicate a negative association. This suggests that practical support for mothers may indeed function differently to emotional or informational support. Nonetheless, with very few studies available, no strong conclusions can currently be made.

An evolutionary anthropological perspective highlights that practical support, from kin members such as grandmothers, may be important factors for maternal breastfeeding. However, these have been generally overlooked in previous studies. The aim of the current study is to build on previous literature by investigating the relationship between practical support from kin members and maternal breastfeeding in the UK Millennium Cohort Study. We identify and incorporate grandmothers as potential sources of kin support, in addition to fathers. As proxies of social support, we use grandmother contact, grandparent financial assistance, father presence, and father’s parental involvement.

## Methods

### Sample

The Millennium Cohort Study (MCS) is an ongoing longitudinal cohort study which covers the whole of the UK. Participants for the MCS were selected from the eligible recruitment pool of children born between 1^st^ September and 31^st^ August 2001 in England and Wales, and children born between 24^th^ November 2000 and 11^th^ January 2002 for Scotland and Northern Ireland. The MCS intentionally oversampled children from particular backgrounds who are often underrepresented in cohort studies, such as children living in disadvantaged areas and ethnic minorities. In total, 18827 children were recruited belonging to 18552 households. The full MCS cohort profile is available elsewhere[40]. We use information collected in the first sweep when the focal children were around 9 months old, which received ethical approval from South West Multicentre Research Ethics Committee. All cases are anonymised, and we did not require additional ethics approval or participant consent for the present study. We remove cases where birthmothers are not present in the household.

### Variables

#### Outcomes: Breastfeeding Initiation and Duration

Information on maternal breastfeeding was retrospectively self-reported by mothers. For breastfeeding initiation, mothers were asked if they had ever tried to breastfeed their child. Therefore, this measure does not necessarily capture breastfeeding success, but an attempt by the mother to initiate breastfeeding. For breastfeeding duration, mothers were asked how old their child was when they last received breast milk, coded into months ranging from 0 to 8+. Note, some mothers were still breastfeeding at the time of the survey. Breastfeeding duration is not limited to exclusive breastfeeding, and includes children who were given formula and solids.

#### Main Predictors: Proxies of Kin Support

As proxies of social support from mother’s partners we use *partnership status* and *father’s parental involvement*. Partnership status is based on co-residence, does not necessarily involve marriage, and is categorised into single mother, father present and stepfather present. This is taken to represent the availability of social support for the mother from her partner, which could be both emotional and practical. Father’s parental involvement is based on the frequency of childcare activities reported by the father, and is only available for father-present households. Fathers were asked how frequently they look after the baby, change the baby’s nappy, and get up at night for the baby, with each activity measured on a scale from 0 (never) to 5 (more than once a day). These were combined to create a parenting score ranging from 0–15, with 15 representing high parental involvement. This is taken to capture the degree of practical support from fathers.

As proxies of social support from grandmothers we use *grandmother contact* and *grandparent financial help*. Information on face-to-face grandmother contact frequency is available separately for maternal and paternal grandmothers. Maternal grandmother contact was reported by the mother, and paternal grandmother contact frequency by the father, meaning information on paternal grandmother contact frequency is only available for father-present households. Contact has been categorised into “lives with”, “daily contact”, “at least once a week”, “at least once a month”, “at least once every few months”, “once a year or less”, and “never” (including grandparent deceased). Given that direct contact is necessary for direct practical support, this is taken as a proxy for practical support.

Information on grandparent financial assistance is available separately for maternal and paternal grandparents. Again, maternal and paternal grandparent support was reported by the mother and father separately, meaning information on paternal grandparent financial assistance is only available for father-present households. The types of financial assistance were wide-ranging, including buying gifts for the baby, contributing to household costs, lending money and paying for childcare. This has been categorised into “any financial assistance” and “no financial assistance”. Financial assistance is viewed as a resource transfer from the grandparents to the mother’s household, taken as a form of practical social support.

#### Controls

We include a variety of covariates previously found to predict breastfeeding duration and initiation, namely country of residence, local indicator of multiple deprivation (measured at ‘Lower Layer Super Output Area’, covering 400 to 1200 households), household income, maternal employment status, number of focal child’s siblings in the household, perceived financial difficulty, home ownership status, maternal education level, paternal education level, mother’s age, child’s ethnicity, child’s sex, birth weight and gestation length.

### Analyses

For breastfeeding initiation we carried out logistic regressions with “1” representing initiation. For breastfeeding duration, due to the right-censored nature of the data we carried out discrete-time event history analyses, which were restricted to mothers who reported breastfeeding initiation. The event represented breastfeeding termination, and the time units were completed months since birth. Note, information on father’s parental involvement and paternal grandmother support is only available for households with a co-resident father. Therefore, we ran full sample analyses with partnership status, maternal grandmother contact and maternal grandparent financial assistance. For a sub sample of father-present households, we included father’s parental involvement, maternal and paternal grandmother contact, and maternal and paternal grandparent financial assistance. All analyses were carried out using STATA/SE v.12.1 (Stata Corporation, Texas, USA), and were weighted using sample weights provided by the MCS in the first wave to account for their stratified clustered design.

## Results

### Characteristics of Study Sample

In the full sample, 66.97% of mothers reported that they had initiated breastfeeding. Of those who initiated breastfeeding, 31.58% of mothers reported breastfeeding for 6 months or more. In the subsample of father-present households breastfeeding rates were slightly higher, with 70.88% of mothers initiating breastfeeding. Of those who initiated, 33.16% mothers reported breastfeeding for 6 months or more. The descriptive statistics for all variables used in the analyses in our full and subsamples are available in Table 1.

**Table 1:** Descriptive statistics for all variables used in the analyses in the study sample.

	Full Sample (N = 16701)	Subsample (N = 10360)		Full Sample (N = 16701)	Subsample (N = 10360)
***Breastfeeding Initiation (%)***			***Partnership Status (%)***		
Yes	66.97	70.88	Single Mother	20.22	—
No	33.03	29.12	Father Present	79.59	—
***Breastfeeding Duration*, *of those who initiated (completed months) (%)***			Stepfather Present	0.19	—
0	23.67	22.39	***Paternal Parenting Involvement***		
1	13.20	12.77	mean	—	8.26
2	9.55	*9*.*46*	(sd)	—	3.31
3	8.95	8.96	range	—	0–15
4	8.17	8.25	***Maternal Employment (%)***		
5	4.88	5.01	Yes	47.19	52.29
6	5.75	6.04	No	52.81	47.71
7	3.46	3.69	***Paternal Employment (%)***		
8+	22.37	23.44	Yes	87.31	87.45
***Maternal Grandmother Contact (%)***			No	12.69	12.55
Lives With	4.84	1.76	***Maternal Education (%)***		
Daily	23.32	22.17	O-level or Equiv.	37.43	35.77
Weekly	35.97	*37*.*86*	A-level or Equiv.	14.11	14.48
Monthly	9.09	10.06	Degree or Equiv.	29.04	33.20
Every Few Months	9.28	10.28	Overseas	3.02	3.14
Yearly or Less	7.16	7.77	None	16.39	13.41
Never	10.33	10.08	***Sex of Child (%)***		
***Paternal Grandmother Contact (%)***			Male	51.37	51.23
Lives With	—	2.43	Female	48.63	48.77
Daily	—	9.43	***Birth Weight (kg)***		
Weekly	—	40.56	mean	3.34	3.36
Monthly	—	14.81	(sd)	0.59	0.59
Every Few Months	—	11.76	range	0.39–7.23	0.39–7.23
Yearly or Less	—	7.65	***Ethnicity of Child (%)***		
Never	—	13.35	White	82.62	83.14
***Maternal Grandparent Financial Assistance (%)***			South Asian (Any)	10.04	11.31
Yes	75.26	75.04	Black (Any)	3.60	2.28
No	24.74	24.96	Other	3.75	3.27
***Paternal Grandparent Financial Assistance (%)***			***Birth Weight (kg)***		
Yes	—	71.21	mean	3.34	3.36
No	—	28.79	(sd)	0.59	0.59
***Financial Difficulty (%)***			range	0.39–7.23	0.39–7.23
mean	2.30	2.19	***Gestation Length (weeks)***		
(sd)	1.00	0.97	mean	39.55	39.56
range	1–5	1–5	(sd)	2.04	2.03
***Household Income (%)***			range	23–42.29	23.42.29
Top 25%	19.37	23.50	***Mother’s Age at Birth (yrs)***		
Middle 50%	52.78	60.94	mean	28.33	29.11
Bottom 25%	27.85	23.50	(sd)	5.97	5.57
***Home Ownership (%)***			range	13–63	13–51
Renting	35.31	27.20	**Multiparity Birth**		
Own Home	58.31	68.45	Yes	4.93	1.48
Other	6.38	4.35	No	95.07	98.52
***Indices of Multiple Deprivation***			***Number of Focal Child’s Siblings in Household***		
mean	3.58	3.91	mean	0.94	0.96
(sd)	2.93	2.94	(sd)	1.08	1.07
range	0–9	0–9	range	0–9	0–9
***Country (%)***					
England	62.16	63.02			
Wales	14.88	14.10			
Scotland	12.60	12.77			
Northern Ireland	10.37	10.11			

### Fathers and Breastfeeding

Table 2 displays the key results for breastfeeding initiation and duration (see SI for full results). In the full sample, compared to single-mothers, mothers in father-present households were 33.6% more likely to initiate breastfeeding (95% CI 1.154, 1.546). No significant association was found between partnership status and breastfeeding duration. In the subsample of father-present households with information on father’s parental involvement, a 1 point increase in father involvement predicted greater odds of breastfeeding termination by 3.6%, which is equivalent to an increase in odds by 12.3% for a 1 SD increase in father involvement (95% CI 1.08, 1.17). No significant association was found between father’s parental involvement and breastfeeding initiation. Overall, these results suggest that the availability of fathers as supporters may be associated with greater breastfeeding initiation, while practical parenting support may be associated with shorter breastfeeding duration.

**Table 2:** Key results of regression models on breastfeeding initiation and termination.

	Breastfeeding Initiation				Breastfeeding Termination (of those who Initiated)			
	Full Sample		Father Present Subsample		Full Sample		Father Present Subsample	
**N(Mothers)**	16701		10360		11146		7515	
	**OR**	**95%CI**	**OR**	**95%CI**	**HR**	**95%CI**	**HR**	**95%CI**
**Partnership Status**								
Single Mother (ref)	—	—	—	—	—	—	—	—
Father Present	1.336***	1.154,1.546	—	—	0.952	0.851,1.064	—	—
Stepfather Present	1.048	0.458,2.40	—	—	0.987	0.470,2.072	—	—
**Father’s Parental Involvement**	—	—	0.984	0.966,1.003	—	—	1.036***	1.024,1.047
**Maternal Grandmother Contact**								
Lives With	0.950	0.726,1.243	0.797	0.498,1.277	0.974	0.806,1.178	0.852	0.609,1.192
Every Day (ref)	—	—	—	—	—	—	—	—
Weekly	1.399***	1.252,1.562	1.478***	1.280,1.707	0.924	0.851,1.003	0.914	0.825,1.012
Monthly	2.187***	1.823,2.624	2.240***	1.771,2.835	0.747***	0.670,0.834	0.764***	0.668,0.873
Every Few Months	2.624***	2.153,3.198	2.601***	2.020,3.350	0.749***	0.671,0.836	0.794***	0.695,0.907
Yearly or Less	2.671***	2.090,3.415	2.848***	2.060,3.939	0.636***	0.550,0.734	0.673***	0.563,0.805
Never	1.451***	1.217,1.730	1.700***	1.343,2.149	0.898	0.793,1.017	0.959	0.821,1.121
**Paternal Grandmother Contact**								
Lives With	—	—	0.765	0.459,1.275	—	—	0.700*	0.519,0.943
Every Day (ref)	—	—	—	—	—	—	—	—
Weekly	—	—	1.093	0.890,1.343	—	—	0.876	0.757,1.014
Monthly	—	—	1.411**	1.103,1.805	—	—	0.875	0.745,1.027
Every Few Months	—	—	1.635***	1.244,2.148	—	—	0.657***	0.556,0.777
Yearly or Less	—	—	1.828***	1.328,2.516	—	—	0.749**	0.620,0.904
Never	—	—	1.285	0.995,1.660	—	—	0.767**	0.646,0.911
**Maternal Grandparent Financial Assistance**								
Yes (ref)	—	—	—	—	—	—	—	—
No	1.050	0.934,1.181	1.088	0.934,1.267	1.005	0.933,1.084	1.043	0.951,1.145
**Paternal Grandparent Financial Assistance**								
Yes (ref)	—	—	—	—	—	—	—	—
No	—	—	1.105	0.958,1.274	—	—	0.938	0.859,1.025

*P≤0.05

**P≤0.01

***P≤0.001

### Grandmothers and Breastfeeding

In general, mothers with higher frequencies of maternal and paternal grandmother contact were associated with lower rates of breastfeeding initiation and shorter duration of breastfeeding. In the full sample, compared to everyday contact, mothers who had contact with maternal grandmothers once every few months were more likely to initiate breastfeeding by 162.4% (95% CI 2.153, 3.198), with lower odds of breastfeeding termination by 25.1% (HR 0.749; 95% CI 0.671, 0.836). In the subsample where information on paternal grandmother contact is available, compared to everyday contact, mothers who had contact with paternal grandmothers once every few months were also more likely to initiate breastfeeding by 63.5% (95% CI 1.244, 2.148), with lower odds of breastfeeding termination by 34.3% (HR 0.657; 95% CI 0.556, 0.777) (Table 2).

The only result which did not fit this trend was cohabitation with paternal grandmothers and breastfeeding duration. Compared to everyday contact, cohabitation with paternal grandmothers was associated with lower odds of breastfeeding termination by 30% (HR 0.700; 95% CI 0.519, 0.943), indicating that mothers who cohabited with paternal grandmothers breastfed for longer. While the reasons behind this finding are unclear, it suggests that there may be unmeasured differences between families who co-reside with paternal grandmothers and other family types. Finally, we found no significant relationship between financial assistance from maternal/paternal grandparents and breastfeeding initiation or duration (Table 2).

## Discussion

### Main Findings

Our results indicate a negative relationship between practical support and breastfeeding in a developed country. First, we find that frequent contact with grandmothers is associated with a lower likelihood of breastfeeding initiation and a higher risk of breastfeeding termination, while father’s parental involvement predicts a higher risk of breastfeeding termination. However, we find that father presence itself is associated with greater odds of breastfeeding initiation. This suggests that support for mothers related to the presence of a father, possibly related to emotional support, may function differently to practical support given by the father. Our previous findings from a similar cohort study showed that father presence is related to higher levels of maternal parenting[41]. One possible interpretation is that father presence serves as an environmental cue which encourages greater maternal investments to optimise child quality. Finally, we found no evidence to suggest that financial assistance influences breastfeeding, which could suggest that support involving direct contact is the important factor regarding practical support for breastfeeding.

The current results are in line with our suggestion that practical support differs from emotional support, and complements the two previous studies which found that contact with maternal grandmothers predicts lower levels of breastfeeding[38,39], with our study based on a substantially larger sample. Speculating on a possible mechanism, frequent contact and practical support may be associated with lower levels of breastfeeding which enables helpers to take a greater part in childcare activities, including infant feeding. While breastfeeding is not a substitutable activity, infant feeding can be substituted through the use of formula. Formula feeding is likely to reduce the dependency of the child on the mother, opening up more opportunities for fathers and grandmothers to assist with direct care activities while allowing mothers to carry out other activities. In support, a qualitative study on British mothers highlights that bottle-feeding can be perceived as a practise which facilitates bonding with other carers such as fathers and grandmothers, stemming from the fact that these allomothers can participate in infant feeding. Furthermore, mothers implicitly reported that bottle-feeding by allomothers substitutes breastfeeding, allowing family members to share the costs of infant feeding, freeing up mothers so they can rest or carry out other activities[42].

While previous literature generally shows a positive association between support and maternal breastfeeding, these tend to focus on emotional and informational support. The current findings imply differences in the types of support mothers may receive, and the associated outcomes on breastfeeding: It highlights the possibility that practical support given to mothers may operate differently from other types of support. Practical support can create incentives for mothers to formula-feed.

From an evolutionary anthropological standpoint, the current findings suggest that support from allomothers such as fathers and grandmothers may influence maternal caregiving behaviour in developed populations such as the UK. Like traditional populations, close kin in developed populations may be important allomothers, perhaps reflecting the maintenance of cooperative breeding within the childrearing systems of low-fertility low-mortality contexts.

### Limitations

Due to the retrospective nature of the information, it is important to note that the causal direction of these associations is not addressed in the current study. It may be that paternal involvement and frequent grandparent contact creates an incentive for mothers not to breastfeed in order to take advantage of the practical support. Equally, it may be that fathers and grandmothers increase practical support when mothers are not breastfeeding, as there is a greater opportunity for them to provide support. Furthermore, while contact is a prerequisite for and surely correlated with direct practical support, the current study is limited by the unavailability of detailed information on the type/intensity of interactions which occur between grandmothers and mothers. Different types of interactions may have different associations with maternal breastfeeding. For example, assisting with household chores should be possible whether mothers are breastfeeding or formula-feeding, while babysitting may be easier for grandmothers if infants are formula-fed.

This also reflects how the relationship between allomother support and maternal breastfeeding is unlikely to be one-directional. While mothers are theorised to require support, suggesting a transfer from the supporter to the mother, the type and intensity of support provided may be influenced by maternal behaviour, and vice versa. For instance, mother-child co-sleeping is known to promote breastfeeding[43], and there is some evidence that father-child co-sleeping is associated with greater paternal parenting[44]. Assuming mother-child co-sleeping may naturally encourage father-child co-sleeping, this maternal co-sleeping behaviour relating to breastfeeding may influence paternal support, which could feed back into maternal breastfeeding.

While the complexities behind practical support and maternal breastfeeding in developed contexts is unclear in the current study, our findings nonetheless highlight a previously overlooked relationship where mothers who breastfeed, and those breastfeed for longer, are likely to receive less practical support from fathers and grandmothers. For future studies, we encourage research into the effects of different kinds of practical support on breastfeeding in conjunction with emotional support, and extend the focus of potential supporters to include grandmothers.

## Supporting Information

S1 Table Full results of breastfeeding initiation and breastfeeding termination models with all covariates. (PDF)
