# Supplementary material for: Practical Support from Fathers and Grandmothers Is Associated with Lower Levels of Breastfeeding in the UK Millennium Cohort Study
Source: PLoS One. 2015 Jul 20;10(7):e0133547. doi: 10.1371/journal.pone.0133547 (PMC4507871; doi:10.1371/journal.pone.0133547)
Supplement: S1 Table — (PDF) [file pone.0133547.s001.pdf]

SUPPORTING INFORMATION

S1 Table 1: Full results of breastfeeding initiation and breastfeeding termination models with all covariates.

	Breastfeeding Initiation				Breastfeeding Termination (of those who Initiated)			
	Full Sample		Subsample		Full Sample		Subsample	
**N(Mothers)**	16701		10360		11146		7515	
	OR	95%CI	OR	95%CI	OR	95%CI	OR	95%CI
**Partnership Status**								
Single Mother (ref)	--	--	--	--	--	--	--	--
Father Present	1.336***	1.154,1.546	--	--	0.952	0.851,1.064	--	--
Stepfather Present	1.048	0.458,2.40	--	--	0.987	0.470,2.072	--	--
**Father Parental Involvement**	--	--	0.984	0.966,1.003	--	--	1.036***	1.024,1.047
Lives With	0.950	0.726,1.243	0.797	0.498,1.277	0.974	0.806,1.178	0.852	0.609,1.192
Every Day (ref)	--	--	--	--	--	--	--	--
Weekly	1.399***	1.252,1.562	1.478***	1.280,1.707	0.924	0.851,1.003	0.914	0.825,1.012
Monthly	2.187***	1.823,2.624	2.240***	1.771,2.835	0.747***	0.670,0.834	0.764***	0.668,0.873
Every Few Months	2.624***	2.153,3.198	2.601***	2.020,3.350	0.749***	0.671,0.836	0.794***	0.695,0.907
Yearly or Less	2.671***	2.090,3.415	2.848***	2.060,3.939	0.636***	0.550,0.734	0.673***	0.563,0.805
Never	1.451***	1.217,1.730	1.700***	1.343,2.149	0.898	0.793,1.017	0.959	0.821,1.121
**PGM Contact**								
Lives With	--	--	0.765	0.459,1.275	--	--	0.700*	0.519,0.943
Every Day (ref)	--	--	--	--	--	--	--	--
Weekly	--	--	1.093	0.890,1.343	--	--	0.876	0.757,1.014
Monthly	--	--	1.411**	1.103,1.805	--	--	0.875	0.745,1.027
Every Few Months	--	--	1.635***	1.244,2.148	--	--	0.657***	0.556,0.777
Yearly or Less	--	--	1.828***	1.328,2.516	--	--	0.749**	0.620,0.904
Never	--	--	1.285	0.995,1.660	--	--	0.767**	0.646,0.911
**MG Financial Help**								
Yes (ref)	--	--	--	--	--	--	--	--
No	1.050	0.934,1.181	1.088	0.934,1.267	1.005	0.933,1.084	1.043	0.951,1.145
**PG Financial Help**								
Yes (ref)	--	--	--	--	--	--	--	--
No	--	--	1.105	0.958,1.274	--	--	0.938	0.859,1.025
**†Mother's Age**	1.037***	1.027,1.047	1.028***	1.015,1.041	0.961***	0.955,0.968	0.960***	0.952,0.968
**†Birth Weight (kgs)**	1.129*	1.025,1.243	1.156*	1.017,1.314	0.937*	0.878,0.999	0.950	0.877,1.028
**†Gestation Length (wks)**	0.989	0.962,1.017	0.980	0.946,1.016	0.967***	0.950,0.984	0.964***	0.943,0.985
**Multiparity Birth**								
No (ref)	--	--	--	--	--	--	--	--
Yes	1.926***	1.311,2.830	1.923**	1.193,3.100	0.645***	0.498,0.836	0.639**	0.474,0.862
**Child Sex**								
Male (ref)	--	--	--	--	--	--	--	--
Female	0.973	0.892,1.063	0.941	0.839,1.055	0.924**	0.873,0.979	0.911**	0.850,0.977
**Child Ethnicity**								
White (ref)	--	--	--	--	--	--	--	--
South Asian	3.303***	2.684,4.065	2.896***	2.161,3.879	0.758***	0.675,0.852	0.861	0.737,1.005
Black	12.658***	8.499,18.853	8.458***	3.961,18.063	0.531***	0.452,0.624	0.565***	0.428,0.745
Other	2.772***	2.123,3.619	3.795***	2.386,6.037	0.669***	0.578,0.774	0.651***	0.538,0.787
**Number of Child's Siblings in Household**	0.763***	0.727,0.802	0.756***	0.710,0.805	0.918***	0.887,0.950	0.903***	0.866,0.943
**Income**								
Bottom 25%	--	--	--	--	--	--	--	--
Middle 50%	1.136	0.993,1.300	1.222*	1.009,1.480	0.970	0.881,1.068	0.985	0.860,1.129
Top 25%	1.444***	1.188,1.754	1.572***	1.222,2.021	1.067	0.945,1.205	1.129	0.962,1.325
**Home Ownership**								
Renting	--	--	--	--	--	--	--	--
Owner	1.206**	1.071,1.359	1.281**	1.097,1.496	0.915*	0.842,0.995	0.861**	0.776,0.956
Other	1.248	0.982,1.585	1.590*	1.089,2.322	0.804**	0.690,0.936	0.708**	0.569,0.881

**P*≤0.05 ***P*≤0.01 ****P*≤0.001 †*mean-centred*

S1 Table 1: Continued.

	Breastfeeding Initiation				Breastfeeding Termination (of those who Initiated)			
	Full Sample		Subsample		Full Sample		Subsample	
	OR	95%CI	OR	95%CI	OR	95%CI	OR	95%CI
**Indices of Multiple Deprivation**	1.080***	1.061,1.100	1.063***	1.039,1.087	0.973***	0.962,0.985	0.979**	0.965,0.993
**Perceived Financial Difficulty**	1.075**	1.025,1.128	1.081*	1.015,1.151	1.025	0.994,1.058	1.014	0.975,1.054
**Mother's Education**								
None	0.615***	0.537,0.703	0.731***	0.603,0.886	1.183**	1.058,1.322	1.202*	1.032,1.401
O-Level or Equiv. (ref)	--	--	--	--	--	--	--	--
A-Level or Equiv.	1.540***	1.353,1.752	1.629***	1.377,1.928	0.734***	0.673,0.800	0.729***	0.655,0.812
Degree or Equiv.	2.760***	2.422,3.145	2.952***	2.510,3.472	0.610***	0.566,0.656	0.598***	0.546,0.654
Overseas	1.306	0.986,1.730	1.298	0.881,1.911	0.748**	0.619,0.903	0.742*	0.584,0.942
**Mother's Employment Status**								
Unemployed (ref)	--	--	--	--	--	--	--	--
Employed	0.965	0.872,1.068	0.884	0.775,1.007	1.246***	1.168,1.329	1.264***	1.167,1.369
**Father's Employment Status**								
Unemployed (ref)	--	--	--	--	--	--	--	--
Employed	--	--	1.166	0.952,1.427	--	--	1.002	0.867,1.158
**Country**								
England (ref)	--	--	--	--	--	--	--	--
Wales	0.780***	0.695,0.876	0.781***	0.672,0.909	1.105*	1.021,1.197	1.067	0.968,1.177
Scotland	0.679***	0.602,0.766	0.650***	0.558,0.757	0.980	0.903,1.063	0.936	0.848,1.034
Northern Ireland	0.486***	0.426,0.554	0.527***	0.440,0.631	1.616***	1.460,1.789	1.520***	1.337,1.728
**Time (Completed Months Since Birth)**								
0 (ref)	--	--	--	--	--	--	--	--
1	--	--	--	--	0.816***	0.749,0.888	0.796***	0.716,0.885
2	--	--	--	--	0.707***	0.644,0.777	0.740***	0.660,0.831
3	--	--	--	--	0.871**	0.790,0.959	0.877*	0.779,0.988
4	--	--	--	--	1.027	0.929,1.136	1.054	0.933,1.191
5	--	--	--	--	0.724***	0.642,0.816	0.792***	0.687,0.913
6	--	--	--	--	1.114	0.992,1.251	1.217**	1.061,1.396
7	--	--	--	--	0.737***	0.641,0.847	0.819*	0.695,0.965
**Constant**	0.309***	0.197,0.487	0.296***	0.155,0.563	0.733*	0.537,0.999	0.620*	0.411,0.936

*P≤0.05 **P≤0.01 ***P≤0.001
